# Efficacy of Modified Atkins Ketogenic Diet in Chronic Cluster Headache: An Open-Label, Single-Arm, Clinical Trial

**DOI:** 10.3389/fneur.2018.00064

**Published:** 2018-02-12

**Authors:** Cherubino Di Lorenzo, Gianluca Coppola, Davide Di Lenola, Maurizio Evangelista, Giulio Sirianni, Paolo Rossi, Giorgio Di Lorenzo, Mariano Serrao, Francesco Pierelli

**Affiliations:** ^1^Don Carlo Gnocchi Onlus Foundation, Milan, Italy; ^2^G. B. Bietti Foundation-IRCCS, Research Unit of Neurophysiology of Vision and Neurophthalmology, Rome, Italy; ^3^Department of Medico-Surgical Sciences and Biotechnologies, “Sapienza” University of Rome Polo Pontino, Latina, Italy; ^4^Istituto di Anestesiologia, Rianimazione e Terapia del Dolore, Università Cattolica del Sacro Cuore/CIC, Rome, Italy; ^5^Delle Medical Center, Wellness and Dietary Medicine, Rome, Italy; ^6^INI, Headache Clinic, Grottaferrata, Rome, Italy; ^7^Laboratory of Psychophysiology, Department of Systems Medicine, University of Rome “Tor Vergata”, Rome, Italy; ^8^INM Neuromed IRCCS, Isernia, Italy

**Keywords:** ketogenic diet, modified Atkins diet, ketogenesis, ketone bodies, cluster headache, drug resistance

## Abstract

**Introduction:**

Drug-resistant cluster headache (CH) is still an open clinical challenge. Recently, our group observed the clinical efficacy of a ketogenic diet (KD), usually adopted to treat drug-resistant epilepsies, on migraine.

**Aim:**

Here, we aim to detect the effect of KD in a group of drug-resistant chronic CH (CCH) patients.

**Materials and methods:**

Eighteen drug-resistant CCH patients underwent a 12-week KD (Modified Atkins Diet, MAD), and the clinical response was evaluated in terms of response (≥50% attack reduction).

**Results:**

Of the 18 CCH patients, 15 were considered responders to the diet (11 experienced a full resolution of headache, and 4 had a headache reduction of at least 50% in terms of mean monthly number of attacks during the diet). The mean monthly number of attacks for each patient at the baseline was 108.71 (SD = 81.71); at the end of the third month of diet, it was reduced to 31.44 (SD = 84.61).

**Conclusion:**

We observed for the first time that a 3-month ketogenesis ameliorates clinical features of CCH.

**Clinical Trial Registration:**

www.ClinicalTrials.gov, identifier NCT03244735.

## Introduction

Cluster headache (CH) is characterized by unilateral trigeminovascular and autonomic system co-activation. The headaches (0.5–8 attacks per day, lasting 15–180 min) persist for weeks or months (bout), followed by a complete remission until the start of the next cluster of attacks. If patients have no remission periods, their CH is defined as chronic (CCH) in contrast to the episodic form (ECH) (1).

Chronic CH remains an open challenge for clinicians; several patients are drug resistant or unsatisfied by current treatments. They, looking for relief, may abandon the medical care and resort to alternative medicines or illicit substances (2).

Our group has observed migraine patients’ headache relief following a ketogenic diet (KD) (3), a nutritional regime usually adopted to treat drug-resistant epilepsies. Afterward, we have prescribed the KD to a migraine patient also affected by drug-resistant CCH and, surprisingly, both headaches responded to the diet. Since some of the mechanisms supposed to be the target of the KD in migraine are shared with CH (such as activation of the trigeminal nerve in mediating pain and monoaminergic involvement) (4), it is of interest to explore whether a KD intervention is effective as a prophylactic treatment for CH. In this open-label study, we therefore aimed to assess the efficacy and the safety of KD in patients with drug-resistant CCH.

## Materials and Methods

### Study Design

A prospective, open-label, single-arm clinical trial.

### Participants

This case series was conducted with the support of the Italian branch of the Organisation for the Understanding of Cluster Headache (OUCH-Italia), which referred to us chronic patients who abandoned the medical care but wished to try the KD. Our local Ethics Committee gave approval for the study, and written informed consent was obtained from all the participants. The trial was registered on ClinicalTrials.gov (NCT03244735). Patients received information about the diet and were asked to complete a headache diary (attack frequency and name/number of acute headache medication) for 1 month before the start of the diet and during the 12-week study period.

The inclusion criteria were diagnosis of CCH according to the ICHD-3 beta criteria (1) and abandonment of previous drug prophylactic treatments (see Results).

The exclusion criteria were regular extra-headache medication intake (i.e., antibiotics, corticosteroids, antidepressants, benzodiazepines, and illicit drugs) except for hormonal contraceptives, history of other neurological diseases, connective or autoimmune diseases, and any other type of primary or secondary headaches. Moreover, for uniformity in the dietetic intervention, we excluded over-weight and obese patients who were referred to a different dietetic regime. Female participants were informed that in case of pregnancy they should drop out of the study. All study participants were unfamiliar with the KD and received a complete description of the study and gave informed consent. The project was approved by the Ethics Committee of the “Sapienza” University of Rome, Polo Pontino and was compliant with the Committee on Publication Ethics and the International Committee of Medical Journal Editors recommendations for ethics.

The responder rate (≥50% reduction of attack frequency) was calculated. Patients were interviewed about adverse events at each monthly visit. An explorative statistical analysis with repeated measures Analysis of Variance (rm-ANOVA) model was adopted to detect differences between the baseline and the other months of diet.

### Modified Atkins Diet

Patients were administered a 12-week ketogenic dietary regime [modified Atkins diet (MAD)] (5) consisting of low carbohydrate (about 15 g/day), normal/low protein (about 0.7–1.2 g/kg/day), and high fat (approximately little more than the weight of carbohydrates and proteins together) from meals prepared by common foods. To summarize, no more than 10 g of carbohydrates per day are allowed in the first month (after that, up to 20–30 g/day) subdivided into three regular-size meals a day or four to five smaller meals. Each meal consists of a liberal combination of fat and protein in the form of fish, shellfish, poultry, red meat, eggs, or low-carbohydrate and high-fat cheese, dressed with butter, heavy whipping cream, mayonnaise, olive oil, and other vegetable oils. Almonds, nuts, and oilseeds are suitable as snack. Salad vegetable twice per day, dressed with oil, mayonnaise or sour cream, vinegar (without added sugars, no balsamic), and salt. Leafy vegetables, up to 200 g per portion. Other vegetables are limited. All spices to add taste (without added sugar) are allowed. Patients must avoid rice, grains, cereals and derivate (bread, pasta, crackers, cookies, etc.), legumes, starchy vegetables (potatoes, corn, green peas), fruits, and dairy products other than cheese, cream, or butter. The exact amount of food per day was suggested to each patient according to their caloric needs (as determined by dietician), but patients could adjust the quantity of foods if required by their sense of satiety/appetite. The caloric intake was possibly adjusted according to patients’ caloric needs, with lipids in the form of a powder composed of medium chain triglycerides, as well as omega-3, and long chain triglycerides (Ketoneural Lipid Complex, Medi-Diet s.r.l., Aprilia, Italy) with nutraceutical integrators (3). Patients were advised to drink 2–2.5 l of water per day. A daily urine stick test confirmed the presence of ketogenesis. Patients reported in the diary each attack, the use of symptomatic treatments, and any eventual side effects or adverse events related to the diet (Appendix S1 in Supplementary Material). Moreover, they had medical supervision for every 4 weeks and laboratory blood tests (alanine aminotransferase, aspartate aminotransferase, gamma glutamic transpeptidase, lactic dehydrogenase, alkaline phosphatase, bilirubin, blood urea nitrogen, and creatinine) at the start and end of the 12-week KD.

## Results

Organisation for the Understanding of Cluster Headache-Italia referred 30 patients to us, of which 19 had CCH, 11 were episodic, and thus excluded from the survey. Out of 19 CCH patients, 18 (11 males; 7 females) elected to take part in the diet after they received explanations and completed the 1-month headache diary. Demographics information and headache characteristics are given in Table 1. The previous prophylactic treatments (reported to be ineffective or not tolerated) included verapamil (240–1,080 mg/day) and lithium (up to 900 mg/day) alone or in combination with other treatments such as sodium valproate (up to 1,200 mg/day), topiramate (up to 400 mg/day), gabapentin (up to 1,600 mg/day), amitriptyline (up to 75 mg/day), and various corticosteroids. Each patient had previously tried at least three different prophylaxis drugs.

**Table 1:** Sociodemographic and clinical characteristics of 18 chronic cluster headache (CH) patients.

Patient ID	Age	CH duration	Chronic from	Attacks for months			
				Baseline	Diet month		
					1st	2nd	3rd
1	26	4	4	63	3	0	0
2	51	22	12	210	208	118	5
3	44	19	7	121	110	58	0
4	39	8	3	59	2	0	0
5	25	6	2	30^c^	2	0	0
6	41	15	5	100	59	28	0
7	47	20	7	211^b^	148	90	10
8	37	8	2	122	61	29	0
9	40	21	5	61	4	0	0
10	30	5	2	30	2	0	0
11	55	30	15	120	58	29	14
12	26	9	3	62	60	59	61
13	42	12	5	93	88	17	28
14	44	22	12	121	63	3	0
15	29	14	9	32	2	0	0
16	45	15	3	91	7	88	93
17	36	8	4	63	4	0	0
18	28	6	6	358^b^	340	360	355
Mean	25–55^a^	13.56	5.89	108.17	67.83	48.83	31.44
SD		7.43	3.83	81.71	89.45	86.11	84.61

*CH duration = duration in years of CH from the first episode*.

*Chronic from = duration in years of chronification of CH*.

*^a^Range of ages*.

*^b^Two patients had more than eight attacks per day occasionally (#7) or daily (#18). They reported that the number of attacks increases as a consequence of the use of sumatriptan s.c. that determined a sort of “shift effect” of attacks (sudden remission of the pain and recurrence within a few hours). Even if it is in contrast to the classification criteria (they apparently could be regarded as paroxysmal hemicrania patients), the response to CH symptomatic therapies (oxygen and sumatriptan) and the lack of response to indomethacin (specific for paroxysmal hemicranias) allowed us to confirm the diagnosis of CH and consider the higher frequency of attacks as a sign of a drug-induced transformation*.

*^c^One patient (#5) experienced attacks with durations >180 min (≅240 min). According to the classification, this patient can be classified as 3.5.1 probable CH*.

**Figure 1:**
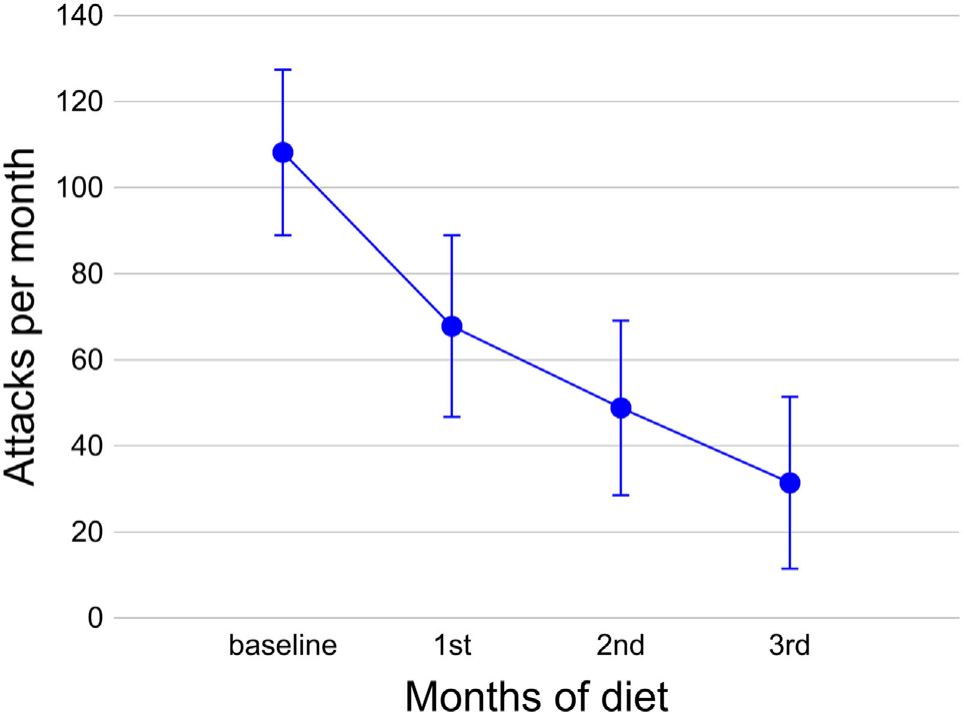
Number of attacks during the diet in 18 chronic cluster headache patients. Circles indicate the mean number of attacks per month at the baseline and during the 3 months of diet; bars indicate the SEM.

Of the 18 CCH patients, 15 were considered responders: 11 experienced a full resolution of headache (6 patients in the first 4 weeks, 2 patients in 8 weeks, and 3 patients in 12 weeks) and 4 had a headache reduction of at least 50% in terms of mean monthly number of attacks. Of the three non-responders, two had no response within the 12-week diet period; one experienced an early response, but had difficulties in maintaining the ketosis for repeated protocol violations, and when he started the diet again, he reported the absence of response. The progressive monthly reduction of CH attacks is summarized in Figure 1.

The explorative analysis with an rm-ANOVA model revealed that the decrease in attacks for months (dependent variable) observed during the diet (within-subject variable: “time,” baseline vs. first month vs. second month vs. third month) was significant (multivariate test: Wilks’ λ = 0.188, *F*_3,15_ = 21.614, *p* < 0.0001; univariate test, adjusted for the absence of sphericity: *F*_3,51_ = 18.392, G-G ε = 0.456, *p* < 0.0001) with a large effect size $\left(\eta_{p}^{2}=0.520\right)$. *Post hoc* tests (with Bonferroni’s confidence interval adjustment for multiple comparisons) of an rm-ANOVA model showed that the decreases in attacks for months observed with respect to the baseline were significant for the first month (*p* = 0.003), second month (*p* < 0.0001), and third month (*p* < 0.0001); the decrease in attacks from the first to second month of the diet was also significant (*p* = 0.009).

As for side effects, one patient reported hair-loss and the other reported abdominal bloating. Biotin and selenium supplementation and activated charcoal and probiotics administration successfully treated both disorders, respectively. None of the patients reported major side effects.

Of the 15 CCH responders, 12 decided to continue the diet over the 12 weeks without interruptions. They are still in the follow-up phase, undergoing neurological supervision every 3 months and laboratory blood tests every 6 months, without a recurrence of bouts. The three CCH patients who decided to stop the KD at the end of the study period had a recurrence of CH (in one case in 7 weeks, in another in 10 weeks, and in the last within 6 months). Two of them started the diet again, with positive responses, whereas the third patient decided not to re-start the diet.

## Discussion

The results of our observations as recorded by their diary and blood tests show that MAD is associated with an attack frequency reduction in CCH patients, in the absence of any relevant side effects.

Only hypotheses can be made about the mechanisms underpinning the positive clinical effects we have observed in this study. In particular, we paid attention to two possible mechanisms of action.

First, KD can induce an increase in brain dopaminergic activity, as measured in the meso-cortical dopaminergic system of mice (6). This datum is relevant to our study because it is known that CH is associated with impaired dopaminergic stimulation (7). Nonetheless, in a previous report about a drug-resistant chronic patient, we observed that treatment with a dopaminergic agonist improved CH and reverted his trigeminal neurophysiological abnormalities (8), typical of the active phase of cluster (9). Interestingly, similar neurophysiological abnormalities are also typical of episodic migraineurs between attacks and, in this kind of patient, KD induced the same neurophysiological changes of dopaminergic stimulation that we observed in CH (4), suggesting shared mechanisms between ketogenesis and dopaminergic stimulation.

Second, ketone bodies (KBs) can increase the GABAergic activity in rat brains (10). As well as in epilepsy, GABA also seems to play a protective role in CH. In fact, it is known that the sodium salt of the GABA-agonist gamma-hydroxybutyric acid (GHB), a drug registered for narcolepsy and alcoholism, is also effective in preventing CH attacks (11, 12). It is worth noting that the GHB is an isomer (sharing the same molecular formula) of beta-hydroxybutyric acid (one of the three KBs), and their similarity could imply that they have very similar biological effects, including the direct GABAergic agonism.

About the dietetic intervention, the choice of the MAD instead of other KD treatments was due to the observation that it is the most adopted ketogenic dietetic regimen in case of adult epileptic patients, because of the higher compliance than classic KD (13). As reported in the Section “Materials and Methods,” to obtain a fine-tuning of diet some patients possibly used a lipid supplementation with a commercial product in powder that also contains MCT triglycerides and omega-3. The use of this product was very limited and extremely variable among patients and diets of each patient, thus we must exclude that the CH improvement observed in our sample was due to the compound. The choice of that product instead of others was made as a result of the lower price and the easy way to obtain it (home delivery).

We do not think that the unexpected outstanding positive results observed were in any way biased by the fact that all patients were referred from a CH support group. In fact, one can suppose that they were more motivated than typical neurologists’ practice patients as our patients were not previously “keto-enthusiast” subjects. On the contrary, most of them were patients who had lost their confidence in doctors because of previous therapeutic failures. We presented them the KD not as a “natural” or “alternative” treatment, but as a medical treatment in which the diet modification self-produced the drugs (the KBs).

The observational nature of our survey represents the main shortcoming of this report. The gold standard in the field of headache research should be a double blind vs. placebo design. However, the non-pharmacological nature of the treatment makes it very difficult to perform such a study, together with the limited number of drug-resistant CCH patients and their urgent need of cure. This is in line with other open-label and case series studies performed on innovative treatment strategies for drug-resistant CCH patients (14, 15). Moreover, the poor reliability of the urine stick in measuring plasmatic KB levels has led us to not perform a correlation with clinical outcomes. Further studies can explore this correlation by a blood test, the gold standard for this kind of analysis.

We are aware that the impact we observe in this study is large, our results need to be taken with caution. In fact, our study population consists of treating refractory CCH patients, not being under medical care at the time of protocol inclusion. In the recent years, several authors already reported a similar effect with other off-label treatments, but their data were not confirmed by other clinicians nor did this lead to a remarkable change in the clinical practice.

## Conclusion

In summary, drug-resistant CH is one of the greatest challenges in headache medicine, and new therapeutic options are welcome. Our observation suggests that, as well as drug-resistant epileptics and migraineurs, ketogenesis also could help CH patients. Although non-conclusive, our preliminary results seem to be promising and further studies are recommended.

## Ethics Statement

The project was approved by the Ethics Committee of the “Sapienza” University of Rome, Polo Pontino and was compliant with the Committee on Publication Ethics (COPE) and the International Committee of Medical Journal Editors (ICMJE) recommendations for ethics.

## Author Contributions

CL has contributed to study concept and design, study coordination and supervision, data acquisition, statistical analysis, interpretation of the data, and drafting and revising the manuscript. GC and DL have contributed to data acquisition, interpretation of the data, and drafting and revising the manuscript. ME has contributed to study concept and design, study coordination and supervision, interpretation of the data, and drafting and revising the manuscript. GS has contributed to study concept and design, study coordination and supervision, data acquisition, and drafting and revising the manuscript. PR has contributed to study concept and design, data acquisition and data analysis, interpretation of the data, and drafting and revising the manuscript. GL has contributed to study concept and design, statistical analysis, interpretation of the data, and drafting and revising the manuscript. MS and FP has contributed to study concept and design, interpretation of the data, and revising the manuscript.

## Conflict of Interest Statement

The authors declare the absence of any commercial or financial relationships that could be construed as a potential conflict of interest. In particular, none of the authors was involved with the Medi-Diet s.r.l. that produces the lipid supplement used in the present study.
