# Supplementary material for: Efficacy of Modified Atkins Ketogenic Diet in Chronic Cluster Headache: An Open-Label, Single-Arm, Clinical Trial
Source: Front Neurol. 2018 Feb 12;9:64. doi: 10.3389/fneur.2018.00064 (PMC5816269; doi:10.3389/fneur.2018.00064)
Supplement: Supplementary file 1 [file Data_Sheet_1.docx]

Appendix 1

**Modified Atkins Diet Cluster Headache Calendar**

*Record bouts, use of sumatriptan or oxygen, ketones and adverse events daily, weight weekly*

Suma: number of doses of sumatriptan 6 mg s.c. per day

O2: number of oxygen treatments (12 L/min lasting at least 15 min)

Ketones: according to urine stick, write none=0, small=1, moderate=2, or large=3

Ad. Ev.: Indicate each adverse event with a number and describe it in the bottom of the diary (e.g.: 1= weakness; 2= nausea; 3= hair loss; etc)

Name: ___________________________ Month: _____________

| Weight | Mon ___  Attacks  Suma:  O2:  Ketones:  Ad. Ev.: | Tue ___  Attacks  Suma:  O2:  Ketones:  Ad. Ev.: | Wed ___  Attacks  Suma:  O2:  Ketones:  Ad. Ev.: | Thu ___  Attacks  Suma:  O2:  Ketones:  Ad. Ev.: | Fri ___  Attacks  Suma:  O2:  Ketones:  Ad. Ev.: | Sat ___  Attacks  Suma:  O2:  Ketones:  Ad. Ev.: | Sun ___  Attacks  Suma:  O2:  Ketones:  Ad. Ev.: |
| --- | --- | --- | --- | --- | --- | --- | --- |
| Weight | Mon ___  Attacks  Suma:  O2:  Ketones:  Ad. Ev.: | Tue ___  Attacks  Suma:  O2:  Ketones:  Ad. Ev.: | Wed ___  Attacks  Suma:  O2:  Ketones:  Ad. Ev.: | Thu ___  Attacks  Suma:  O2:  Ketones:  Ad. Ev.: | Fri ___  Attacks  Suma:  O2:  Ketones:  Ad. Ev.: | Sat ___  Attacks  Suma:  O2:  Ketones:  Ad. Ev.: | Sun ___  Attacks  Suma:  O2:  Ketones:  Ad. Ev.: |
| Weight | Mon ___  Attacks  Suma:  O2:  Ketones:  Ad. Ev.: | Tue ___  Attacks  Suma:  O2:  Ketones:  Ad. Ev.: | Wed ___  Attacks  Suma:  O2:  Ketones:  Ad. Ev.: | Thu ___  Attacks  Suma:  O2:  Ketones:  Ad. Ev.: | Fri ___  Attacks  Suma:  O2:  Ketones:  Ad. Ev.: | Sat ___  Attacks  Suma:  O2:  Ketones:  Ad. Ev.: | Sun ___  Attacks  Suma:  O2:  Ketones:  Ad. Ev.: |
| Weight | Mon ___  Attacks  Suma:  O2:  Ketones:  Ad. Ev.: | Tue ___  Attacks  Suma:  O2:  Ketones:  Ad. Ev.: | Wed ___  Attacks  Suma:  O2:  Ketones:  Ad. Ev.: | Thu ___  Attacks  Suma:  O2:  Ketones:  Ad. Ev.: | Fri ___  Attacks  Suma:  O2:  Ketones:  Ad. Ev.: | Sat ___  Attacks  Suma:  O2:  Ketones:  Ad. Ev.: | Sun ___  Attacks  Suma:  O2:  Ketones:  Ad. Ev.: |
| Weight | Mon ___  Attacks  Suma:  O2:  Ketones:  Ad. Ev.: | Tue ___  Attacks  Suma:  O2:  Ketones:  Ad. Ev.: | Wed ___  Attacks  Suma:  O2:  Ketones:  Ad. Ev.: | Thu ___  Attacks  Suma:  O2:  Ketones:  Ad. Ev.: | Fri ___  Attacks  Suma:  O2:  Ketones:  Ad. Ev.: | Sat ___  Attacks  Suma:  O2:  Ketones:  Ad. Ev.: | Sun ___  Attacks  Suma:  O2:  Ketones:  Ad. Ev.: |

Adverse events:
